# “They don’t have the history and the stature:” examining perceptions of Caribbean offshore medical schools held by Canadian medical education stakeholders

**Published:** 2018-07-27

**Authors:** Jeffrey Morgan, Valorie Crooks, Jeremy Snyder, John Pickering

**Affiliations:** 1 Department of Geography, Simon Fraser University, British Columbia, Canada; 2 Faculty of Health Sciences, Simon Fraser University, British Columbia, Canada

## Abstract

**Background:**

Caribbean offshore medical schools are for-profit, private institutions that provide undergraduate medical education to primarily international students, including from the United States or Canada. Despite the growing role that offshore medical schools play in training Canadian physicians, little is known about how these institutions are perceived by those in professional and decision-making positions where graduates intend to practice.

**Methods:**

The authors interviewed 13 Canadian medical education stakeholders whose professional positions entail addressing the medical education system or physician workforce. Participants were employed in academic, governmental, and non-governmental organizations in leadership roles.

**Results:**

Thematic analysis revealed three cross-cutting perceptions of offshore medical schools: (a) they are at the bottom of an international hierarchy of medical schools; (b) they are heterogeneous in quality of education and student body; and (c) they have a unique business model, characterized by profit-generating and serving international students.

**Conclusion:**

Consistent growth of the offshore medical school industry in the Caribbean may result in adverse reputational harms for well-established offshore or regional medical schools. Both comparative (e.g., USMLE pass rate) and intuitive factors (e.g., professional familiarity) informed participants’ perceptions. Participants believed that core principles of social accountability in medical education are incompatible with the offshore medical school model.

## Introduction

The Caribbean region is home to a disproportionate number of medical schools (per unit population) compared to global norms.^1^ At the time of writing, the World Directory of Medical Schools lists 56 medical schools operating in the region.^2^ While some serve local and regional populations (e.g., University of the West Indies), most medical schools in the Caribbean serve primarily international students. These private, for-profit schools in the Caribbean that overwhelmingly attract international students are commonly referred to as “offshore” medical schools. Offshore medical schools teach United States (US)-based curricula, target students from countries outside the Caribbean region (e.g., US, Canada, India, Nigeria), and their graduates often intend to return or migrate to the US or Canada for post-graduate medical training, and ultimately to practice.^3-7^ Furthermore, undergraduate clinical training often takes place in the US due to limited capacity in the Caribbean.^7-9^ The offshore medical school industry has grown substantially in recent decades; for example, 20 new offshore medical schools have opened since 2007, representing 36 percent of medical schools currently operating in the Caribbean.^2^ In turn, this industry is quickly transforming the landscape of global medical education.

The growth of the Caribbean offshore medical school industry has occurred alongside an increase in the number of Canadian- and US-citizens choosing to studying medicine abroad. While there are many reasons to study medicine abroad (e.g., family connections, seeking an international experience), most Canadian- and US-citizens do so because of growing competition for entry into domestic medical schools, and rising admission requirements.^6,8,10^ Demand for Canadian medical education far exceeds supply, and some students who are not admitted to medical schools in Canada choose to enroll in medical schools abroad, including the Caribbean. Indeed, the Canadian Resident Matching Service (CaRMS) estimated that Canadians studying medicine abroad enroll in Caribbean offshore medical schools more than anywhere else in the world.^6^

Despite the growing role that offshore medical schools play in training Canadian- and US-citizens, little empirical research exists on the topic, particularly in the Canadian context. Specifically, how these offshore institutions are perceived by those in professional and decision-making positions where offshore medical school graduates intend to practice remains a considerable knowledge gap. This is important given that most Canadians studying abroad ultimately intend on returning to Canada to practice,^6,10,11^ and that perceptions of the quality of their international education by Canadian medical school stakeholders (e.g., individuals whose professional positions entail addressing the medical education system or physician workforce) may determine their ability to practice at home upon graduation. Thus, the current exploratory analysis uses qualitative semi-structured interviews to better understand how Caribbean offshore medical schools are perceived by key medical school stakeholders in Canada.

## Methods

We were guided by case study methodology to deepen our knowledge of how offshore medical schools are understood in the Canadian context. The current analysis complements other case studies we have conducted on the topic using other datasets,^12^ a hallmark of case study methodology.^13^ Study participants held professional positions within the Canadian medical education system (e.g., Canadian universities) or the physician workforce (e.g., associations and non-governmental societies). Our sample included deans and vice-deans of medical schools (n=7), health services researchers who have studied these schools (n=2), and those employed in non-profit societies and associations whose missions are directly related to the Canadian physician workforce (n=4). In turn, we consider our sample to be comprised of stakeholders on the topic of Canadian medical education.

Participants were drawn from six Canadian provinces with two based in the US with specific expertise relevant to the study goal. They were recruited through purposive sampling; contact information was retrieved through publicly available information online. We identified participants through web searches and published reports, and we sought to create representation from across the country. Recruitment ended when no new organizations or participants could be identified. To achieve confidentiality, identifying information related to participants or their affiliations was removed from this article. This research was approved by Simon Fraser University’s Research Ethics Board (2016s0094, approved 8 March 2016). Informed consent was obtained prior to the interviews.

We conducted thirteen 30-90 minute semi-structured interviews by phone or teleconference in May-July 2016. Participants were asked to speak based on their knowledge and first-hand observations and were not required to speak on behalf of the organizations they represented. Collectively, they had many decades of employment in the Canadian medical education or affiliated sectors, and had seen or communicated with thousands of medical students, residents, and health professionals in Canada and beyond. Some had visited offshore medical schools or knew instructors working at such institutions. Participants drew on their own extensive expertise to discuss their professional backgrounds, Caribbean offshore medicals, and the relationship between these schools and the Canadian health care and medical education systems. All interviews were transcribed verbatim upon completion of data collection. At least two authors reviewed each transcript. Following independent review, two meetings were held to discuss the transcripts, identify emerging themes, and prepare for thematic analysis.

## Results

The thirteen participants in our study had varying levels of awareness about Caribbean offshore medical schools, including one participant who disclosed having a friend who had attended such a school. Overall, using thematic analysis we identified three cross-cutting perceptions of Caribbean offshore medical schools brought forward by participants: (a) they are at the bottom of an international hierarchy of medical schools based on perceived quality of education; (b) they are heterogeneous in quality of education and student body; and (c) they have a unique model of medical education, characterized by profit-generating and serving international students. In the remainder of this section we examine these themes, including direct quotes to enhance analytic reliability.

### Hierarchy of quality of education

Participants discussed international medical education in geographic terms, and perceived the quality of education of a medical school as tied to a particular nation or region. In addition to the Caribbean, participants also spoke broadly about schools located in the United Kingdom (UK), Ireland, Eastern Europe, Australia, Israel, South Africa, and Mexico. Participants used these countries or regions to make comparisons to Caribbean offshore medical schools, and characterized the Caribbean as falling at the bottom of a global hierarchy of medical training:

I’d probably trust Australia better than I would trust a Caribbean school. Poland, Hungary, I don’t know…Irish schools are okay, but I would probably trust the Australian schools the most.

I have far less concerns when I know I am working with a resident who has trained at a British School, an Australian school, and to some extent a South African school. After that my biases will begin to show.

This perceived hierarchy positioned Ireland, the UK, and Australia as having the highest quality medical schools, and the Caribbean as having among the lowest quality.

Participants’ perceptions of the quality of education abroad were informed by the several factors. Concerns related to Caribbean offshore medical education included: poor faculty/teacher-to-student ratio; low rates of licensing exam pass rates; absence of research programs; ambiguous or voluntary regional accreditation; and an overall poor quality of education. These concerns were compounded by the fact that many offshore medical schools are relatively new, some without a graduating class:

The schools…in the UK, Ireland, Australia, we know people from those schools, we have research collaborations with those schools…There have been so many new and emerging schools in the Caribbean, they don’t have the history and the stature…so there’s a perception that they’re weaker schools.

As shown here, participants’ concerns about quality of education were driven in part by the lack of awareness and first-hand familiarity with graduates or medical faculty from these institutions. These concerns informed many participants’ perceptions of the hierarchy of institutional quality to which Canadians studying medicine abroad must be attentive.

### Heterogeneity in quality of education and student body

Although many participants were comfortable discussing Caribbean offshore medical schools in broad, geographic terms, they recognized that diversity existed between schools and among students. For example, despite concerns regarding quality of training offered at offshore medical schools, participants saw heterogeneity as an important element to consider when assessing quality of education in the Caribbean region:

I don’t think there’s any question that they’re heterogeneous…the quality is markedly different…the heterogeneity in the quality of Caribbean medical schools is likely greater than the heterogeneity in Canadian medical schools.

In addition to quality of education, some participants also noted heterogeneity among the students enrolled in certain offshore medical schools:

The ones like St. George’s, and Ross, and AUC [American University of the Caribbean] are probably getting…the students who just didn’t get into US [and Canadian] medical schools… Some of the other smaller ones are…taking people who…didn’t take the MCAT [Medical College Admission Test]…who come from India and other places in the world, go to medical school in the Caribbean and then use it as a pathway to get into the US.

In this way, the quality of the medical school was a reflection of which students they could attract. Participants believed students from the US and Canada enrolled in top offshore medical schools, while students from India and elsewhere attended “*other, smalle*r” offshore medical schools. These perceptions were informed by personal and professional experiences:

Well there’s a few solid schools…St. Matthews, St. George’s and Saba. Those are the ones that I’m aware of…it’s more just because I have contacts.

The Caribbean is…for instance, St. George’s is an excellent school, with an excellent reputation…and yet they’re kinda tarred…I don’t know each and every Caribbean school…there are excellent physicians who have come out of Caribbean schools.

These quotes illustrate that personal factors such as a participants’ professional contacts or positive interactions with offshore medical school graduates help to shape held perceptions towards an individual school.

### Unique model of medical education

Participants understood the Caribbean offshore model of medical education to be unique compared to Canadian medical schools, and other international medical schools that Canadian and US-citizens attend (e.g., Ireland). Participants pointed to two key factors that set offshore medical schools apart from other models of medical education: (1) they serve an almost exclusively international student body; and (2) they are not integrated into the communities in which they are located, both in terms of training students for local practice or for offering local residency placements:

They’re obviously not training for their own populations. They’re training more than their need for their own populations…They’re obviously just in it for a particular set of reasons, but they’re not first and foremost about training physicians for their own people.

It’s unique in that they’re making some of the students do their USMLEs [US Medical Licensing Examinations]. That’s different from schools in other parts of the world because they’re not setting up for the American system [in these other regions], they’re for their own country’s system [where the school is based].

The Caribbean schools are built on the model of accepting an enormous number of students…doing no research whatsoever… and having no clinical relations whatsoever… I have visited personally only the ones in Israel, and the ones in Israel are well-established medical schools, full of Israeli students, that have a separate [smaller] channel for the Americans…they do their clinical clerkships in Israel.

Participants regularly contrasted offshore medical schools’ international student bodies to medical schools elsewhere, which “*first and foremost*” were about training physicians for local practice. For example, juxtaposing the Caribbean model with that of Israeli medical schools, which primarily serve Israeli students.

Given that offshore medical schools attract primarily international students, and that there are a number of medical schools (both regional and offshore) already operating in the region, participants assumed that the large majority of offshore medical school graduates never practice in the Caribbean. As opposed to training physicians to practice in the Caribbean region, some participants believed offshore medical schools did not ultimately serve the interests of the Caribbean communities in which they are located, which jeopardized their commitment to social accountability:

I suspect most of their motives aren’t well aligned with the social accountability commitment that medical schools in Canada and the US have made…medical schools have identified…a requirement to give back to the community, and promote health equity…addressing the needs of the most vulnerable…I believe that that those [offshore] schools are just trying to get their students through the USMLE.

These quotes highlight that participants used norms of medical education in the US and Canada, such as primarily serving a local population and social accountability, to explain the perceptions they held about the Caribbean offshore model of medical education and offshore medical schools.

## Discussion

This qualitative thematic analysis has examined how Caribbean offshore medical schools are perceived by Canadian medical school stakeholders. Qualitative methods are useful for hypothesis generating and exploratory analyses,^14^ which is appropriate given the lack of empirical research on the topic and existing knowledge gaps. We believe that the perceptions of these stakeholders are significant for several reasons: First, their perspectives expose hegemonic attitudes about Caribbean offshore medical schools, as participants were largely part of the medical education “establishment” in Canada. Hegemonic beliefs and perceptions work towards legitimizing or normalizing certain ideas and values, and thus play an important role in shaping the discourse of a subject.^15^ Second, as the stakeholders interviewed are in policy-influencing positions, this analysis provides new insight into how issues that pertain to Caribbean offshore medical schools–including the Canadians they graduate–are understood by those who are driving the relevant policy conversations. Finally, these findings can help inform prospective students’ decision-making with regard to studying medicine abroad, and spark dialogue among Canadian medical educators about the practical implications of the perceptions shared here.

Based on participants’ perceptions, students interested in studying medicine abroad may wish to consider the regional hierarchy of quality in medical education discussed in the findings should they wish to compete for clinical placements, and ultimately practice in Canada. Although here we do not challenge the accuracy of this hierarchy, its existence is certainly something worthy of future research. While participants acknowledged heterogeneity among schools, and perceived several Caribbean offshore medical schools to be of high quality and with good reputations, the tendency to discuss medical schools in regional terms risks obfuscating and conflating these important differences that exist across scales. For example, between offshore medical schools, relevant differences exist in their histories, such as the number of years the school has been operational (including whether a school has had a graduating class), and student outcomes, such as the attrition rate and employment outcomes. On the national scale, offshore medical schools operate in different social, economic, and political contexts (e.g., tax concessions, local worker availability). These relative strengths and weaknesses will undoubtedly inform students’ decisions to enroll in an offshore medical school, and may have broad implications when they are conflated.

The consistent growth of the offshore medical school industry in the Caribbean could result in adverse reputational harms for well-established offshore (e.g., St. George’s) or regional medical schools (e.g., the University of the West Indies). This reputational harm is clearly reflected by one participant’s comment: “*they’re all [Caribbean medical schools*] *kinda tarred…I don’t know each and every Caribbean school*.” As new offshore medical schools continue to open (and close) every year,^4^ we believe the Caribbean as a whole, and students who graduate from these institutions, risk becoming increasingly tied to this negative perception, which also feeds notions of a regional hierarchy of medical education. Importantly, future research should adopt critical approaches and methods (e.g., critical discourse analysis) to evaluate the accuracy of the perceived hierarchy presented here, and consider how it may be informed by pre-existing stereotypes and biases of the Caribbean region.

Participants discussed several factors that informed their perceptions of quality at offshore medical schools. Some of these factors were comparative in nature while others were more personal. Comparative factors were those that involved measuring the performance of these schools against one another, or against schools elsewhere. For example, participants commonly brought forward admission standards or licensing exam pass rates as comparative factors informing their perceptions of offshore medical schools. Concerns regarding these pass rates and admission standards have been discussed elsewhere in the literature. For example, van Zanten & Boulet (2011) found that USMLE “step one” first-attempt pass rates varied considerably among Caribbean counties and over time.^16^ Another comparative factor that informed stakeholders’ perceptions of Caribbean offshore medical schools were whether institutions were recognized by medical boards in locations where graduates will seek to practice (e.g., certified by the Medical Board of California).^4,17^

Participants also brought forward personal factors to explain or justify the perceptions they held about Caribbean offshore medical schools. Such factors are relational, informed by one’s social positioning, and dependent on the experiences of the observer. For example, multiple participants mentioned personal and professional familiarity as evidence to support their perceptions. Research activities at training institutions was intuitively and personally understood as a signal of educational quality, while the absence of research stood out as a concern. The absence of research programs at Caribbean offshore medical schools has been problematized elsewhere in the literature,^4,9,18^ and thus participants are not alone in this concern. Some participants indicated that the lack of research engagement at offshore institutions is preventing those in the international medical education community from gaining familiarity with the quality and nature of training they offer through opportunities for research collaboration. Similarly, lack of personal on-site exposure to these institutions left participants to rely heavily on second-hand information and perceptions in understanding Caribbean offshore medical schools. This finding suggest that offshore medical schools could leverage personal or professional relationships, including through research and collaboration, to improve perceptions of quality. That said, little is known about the mechanisms through which such unsubstantiated personal and professional factors ultimately shape stakeholders’ perceptions of these schools both in Canada and elsewhere. Additionally, these personal factors and hegemonic perspectives are inseparable from the racialized context and colonial history of the Caribbean region, which provides important framing for how Western medicine is understood in the region and the hierarchical, gendered, and racialized ways in which it is practiced. This is an important avenue for future research because as one participant said, “*my biases will begin to show*” in relation to how graduates of these schools are understood upon return to Canada.

Participants’ perceptions of social accountability in medical education were clearly grounded in place, and emphasized a commitment to the local communities within a defined geographic region where medical schools were located. This model of social accountability is reflected elsewhere.^19-22^ For example, the Global Consensus for Social Accountability of Medical Schools (2010) emphasizes that social accountability “recognizes the local community as a primary stakeholder” and in doing so, “shares responsibility for a comprehensive set of health services to a defined population in a given geographic area, consistent with values of quality, equity, relevance, and efficiency[…].”^22^ These core principles of social accountability in medical education are incompatible with the offshoring model reported here. For example, the Caribbean offshore model is spatially diffuse. Students generally spend two years learning basic sciences in the Caribbean, while clinical training occurs in the US. Although such clinical experiences may be favourable for students who intend to return to Canada or the US, they clearly do not benefit the Caribbean communities where these schools are based. In addition to clinical learning, most offshore medical schools also have head offices in the US and Canada,^23^ which reflects the fact that many offshore medical schools are foreign-owned and operated from outside the Caribbean. In 2014, for example, St. George’s University received a US$750 million investment from a Canadian private-equity firm that now holds a majority stake in the university.^24^ This ownership model has clear implications for social accountability in the Caribbean, as offshore medical schools are accountable first to foreign shareholders. The findings of this analysis point to the fact that this model also has implications for how these schools are perceived by those in the international medical education community, and specifically for how the quality of education is understood. That said, the perspectives of Caribbean medical education stakeholders are outside the scope of this analysis. These perspectives will be important to capture in future research to fully understand how these schools conceptualize of their relationships to the communities in which they are located.

### Conclusion

Offshore medical schools are for-profit, private institutions in the Caribbean that primarily serve international students who intend to leave the region to practice medicine. This model of medical training has seen rapid growth in recent decades and has changed the landscape of international medical education. Using qualitative interviews, we examined perceptions held by Canadian medical school stakeholders related to offshore medical schools and the Canadians they train. Three cross-cutting themes were brought forward by participants: first, international medical education was discussed in geographic terms, comparing Caribbean schools to those in the UK and Ireland, Australia, and elsewhere. This revealed a perceived international hierarchy of medical education, where Caribbean offshore medical schools were considered to be of relatively poor quality. Second, despite viewing medical schools in regional terms, participants recognized heterogeneity between offshore medical schools. Finally, participants understood the offshore model of medical education to be unique, reflected in its focus on serving international students. Among other concerns, participants suggested this model of medical education could impact offshore medical schools’ commitment to social accountability.
